# Falls and fear of falling among Israeli community-dwelling older people: a cross-sectional national survey

**DOI:** 10.1186/s13584-021-00464-y

**Published:** 2021-04-02

**Authors:** Dvora Frankenthal, Mor Saban, Dolev Karolinsky, Miri Lutski, Shelley Sternberg, Iris Rasooly, Irit Laxer, Inbar Zucker

**Affiliations:** 1 Israel Center for Disease Control (ICDC), Ministry of Health, Ramat-Gan, Israel; 2 Israel Ministry of Health, Division of Geriatrics, Jerusalem, Israel; 3 School of Public Health, Sackler Faculty of Medicine, Tel Aviv University, Tel Aviv, Israel

**Keywords:** Falls, Fear of falling, Geriatrics, Israel, Older people

## Abstract

**Background:**

Falls and fear of falling are a major problem for older people and a leading cause of functional decline and institutionalization. There is limited data on the prevalence of falls in a 12-month period among Israeli older adults. Our main objective was to evaluate the prevalence of falls among Israeli community-dwelling older people aged ≥65 years and to identify factors associated with falls and fear of falling.

**Methods:**

A national cross-sectional interview survey was conducted between February 2018 and April 2019 by the Israeli Center for Disease Control. The prevalence of falls was assessed by asking participants about falling within the 12 months prior to the survey. Fear of falling was assessed by asking participants about the fear of future falls. Multivariate analysis was used to identify factors associated with falls and with fear of falling.

**Results:**

From 5281 households that were eligible for inclusion in this study, 3242 participants (61.4%) completed the survey. Falling at least once in the past year was reported by 23.8% of the respondents and fear of falling by 48.2%. The majority of the participants (91.1%) reported that they had never received any instruction about fall prevention from their medical care provider. In the multivariate analysis, falls and fear of falling were each a risk factor for the other; and were also significantly associated with female gender, major functional difficulties, the use of walking aids, cardiac disease, diabetes mellitus and psychotropic medications.

**Conclusion:**

The prevalence of falls and fear of falling among Israeli community-dwelling older people is comparable to the rates published in other countries. Efforts should be made to increase awareness about falls and their health consequences among older people. The development of specific interventions to target those at higher risk for falls and fear of falling is strongly recommended.

## Introduction

Falls are a major problem for older adults and a leading cause of injury-related death, functional decline and early entry into residential care [1, 2]. A fall is defined by the World Health Organization (WHO) as an event that results in a person coming to rest inadvertently on the ground or floor, or other lower level [3]. About 20–40% of the population aged 60 years or over have been described as reporting at least one fall in a year [4–6]. The etiology of falls is multifactorial and interventions that focus on risk factor reduction are important to prevent falls among older persons [7]. Among the most important consequences of falls are the psychological ones which include a fear associated with falls. This can lead to disability and loss of independence [8].

Fear of falling (FOF) is defined as a concern about falling, and is accompanied by loss of balance, loss of confidence and avoidance of activities [9]. FOF usually arises after a fall but can also present without a history of falls [10]. Great variability in the prevalence of FOF has been reported, ranging from 3% to as high as 85% of community-dwelling older fallers [8, 10].

On November 2017, the Israeli Ministry of Health (MOH) initiated a national program with the aim of reducing the incidence of falls in the older population. The current survey was conducted within the framework of this program. There is limited data on the prevalence of falls in a 12-month period among Israeli Jewish and Arab community-dwelling older adults. The primary objective of the survey was to assess the prevalence of falls among Israeli community-dwelling older adults aged ≥65 years. The aim was to provide primary data of the national rates of self-reported falls that would serve as a baseline for future evaluations of the effectiveness of this national program. A secondary objective was to investigate factors associated with falls and FOF. Identifying risk factors for falls and at-risk populations might help policy makers to develop specific interventions and prevention strategies to target those at highest risk.

## Methods

### Survey design and population

A national cross-sectional telephone interview survey of households in Israel was conducted between February 2018 and April 2019 by the Israeli Center for Disease Control. A random sample of telephone numbers (mobile and landline) of Jewish and Arab households was extracted. The sample was proportionate to the population geographic distribution. Households were considered non-eligible and were excluded from the sample if they fulfilled at least one of the following criteria: there was no resident older than 65 years, the residents did not speak Hebrew or Arabic, the residents were unable to complete the questionnaire due to mental or physical disability, and the telephone line was for business or was disconnected. Households were identified as non-respondents (with unknown eligibility) after 8 failed attempts to make contact. Nonresponses included outright refusals to participate, partially completed interviews, and repeated postponements.

### Questionnaire and data collection

Information was collected by means of a structured questionnaire, using a computer-assisted telephone interview (CATI) system. The questionnaire was administered over the telephone in Hebrew and Arabic by trained interviewers from the corresponding population groups. Information collected by the questionnaire included demographic characteristics, health status (comorbidities, medication use, vision and hearing difficulties), physical activity, functional difficulties (physical and cognitive) and falling-related questions (falls in the preceding year, place of fall, fall-related injuries, fear of falling). The Hebrew questionnaire was translated into Arabic and translated back to Hebrew for quality control. The Hebrew and Arabic questionnaires were pretested on a pilot sample to ensure that the survey was understood by all the respondents.

### Definitions

All the variables were based on self-report. The definitions of key variables for the analysis were based on survey questions.

### Falling and fear of falling

The primary outcome measure was the rate of falling, defined as the presence of one or more self-reported falls in the 12 months prior to the survey. This was assessed by asking participants ‘Have you fallen during the previous year?” (yes/no). If the response was “don’t know” or “don’t remember”, the interview was excluded from the data analysis. If the response was positive, the respondent was asked, “In the past 12 months, how many times did you fall?” In addition, participants were asked if they had ever received instructions about fall prevention from their medical care provider. The secondary outcome measure was FOF, assessed by asking all participants, those who had fallen and those who had not, “Are you afraid of future falling?” (yes/no). If the response was positive, the respondent was asked, “Due to fear of falling, have you stopped doing certain activities you liked or used to do?” (yes/no).

### Variables

We used previous research and expert advice from the Ministry of Health geriatric division to determine which variables were associated with falling and FOF among older people [10, 11]. These variables included demographic characteristics, health status and functional limitations. Chronic diseases were assessed by asking the participant, “Has a doctor ever told you that you had any of the following health conditions: a history of heart attack, cardiac disease, a history of stroke, diabetes, Parkinson’s disease, arthritis, depression or anxiety?”. Daily medicine intake was also self-reported and was assessed by asking “How many daily medications do you usually take (excluding vitamins or dietary supplements)?”. The use of sleeping medications and mood medications was assessed by asking “Do you usually use sleeping medications?” and “Do you usually use medications to improve your mood?”. These were multiple-choice questions with 3 possible answers (“yes always”, “yes sometimes”, “never”). For the current analysis, these 3 categories were grouped into 2 categories (yes/no). Functional status was assessed by asking participants “Do you have any difficulties in dressing or bathing?” and “Do you have any difficulties in doing household activities such as cleaning and shopping?” and “Do you have any difficulties in memory or concentration?” These were multiple-choice questions with 4 possible answers (no difficulty, little difficulty, major difficulty, and no ability to do such activities). The latter two categories were grouped into one for the analysis. Vision difficulties were assessed by the question: “Do you have vision problems?” (yes/no). Hearing difficulties were assessed by 2 questions: “Do you use a hearing aid?” (yes/no) and “Do you have hearing difficulties?” (yes/no). Hearing difficulties were defined as a positive answer to at least one of these questions. Physical activity was assessed by asking “Do you usually do any sports or physical activity in your free time to keep yourself fit and healthy?” (yes/no).

### Sample size

The prevalence of falls among the elderly in accordance with the literature was estimated as 30% [4–6]. The margin of error and confidence level were defined as 2.4 and 95% respectively. The minimum recommended sample size to meet this criterion was 3000 participants. The sample size was calculated using Epi Info, an open source calculator [12].

### Statistical analysis

Data were analyzed using SPSS version 25 (SPSS Inc., Chicago, IL, USA). For the descriptive analysis, we calculated the prevalence and percentages for all variables. The percentages of variables were weighted for population groups, age and gender. The Pearson’s Chi-square test was used to compare categorical variables between those who reported a fall in the preceding year and those who did not. Bonferroni corrections were made for multiple comparisons. Multivariate analysis using a backward stepwise logistic regression model was applied to examine risk factors associated with falls and FOF. The following variables were entered in the multivariate logistic regression model: sociodemographic factors (population group, age, gender and education), health-related factors (comorbidities and the use of medications), cognitive and physical difficulties, physical activity and fall-related factors. A *P* value of < 0.05 was considered statistically significant.

### Ethics statement

According to Israeli legislation, telephone health surveys, as described herein, are conducted within the regulatory capacity of the Israeli Center for Disease Control and do not require an approval of an ethics committee. Therefore, approval of an ethics committee was not needed for this data collection and analysis. Oral informed consent for each participant was obtained after a brief explanation about the health survey, including the objectives and importance.

## Results

**Fig. 1:**
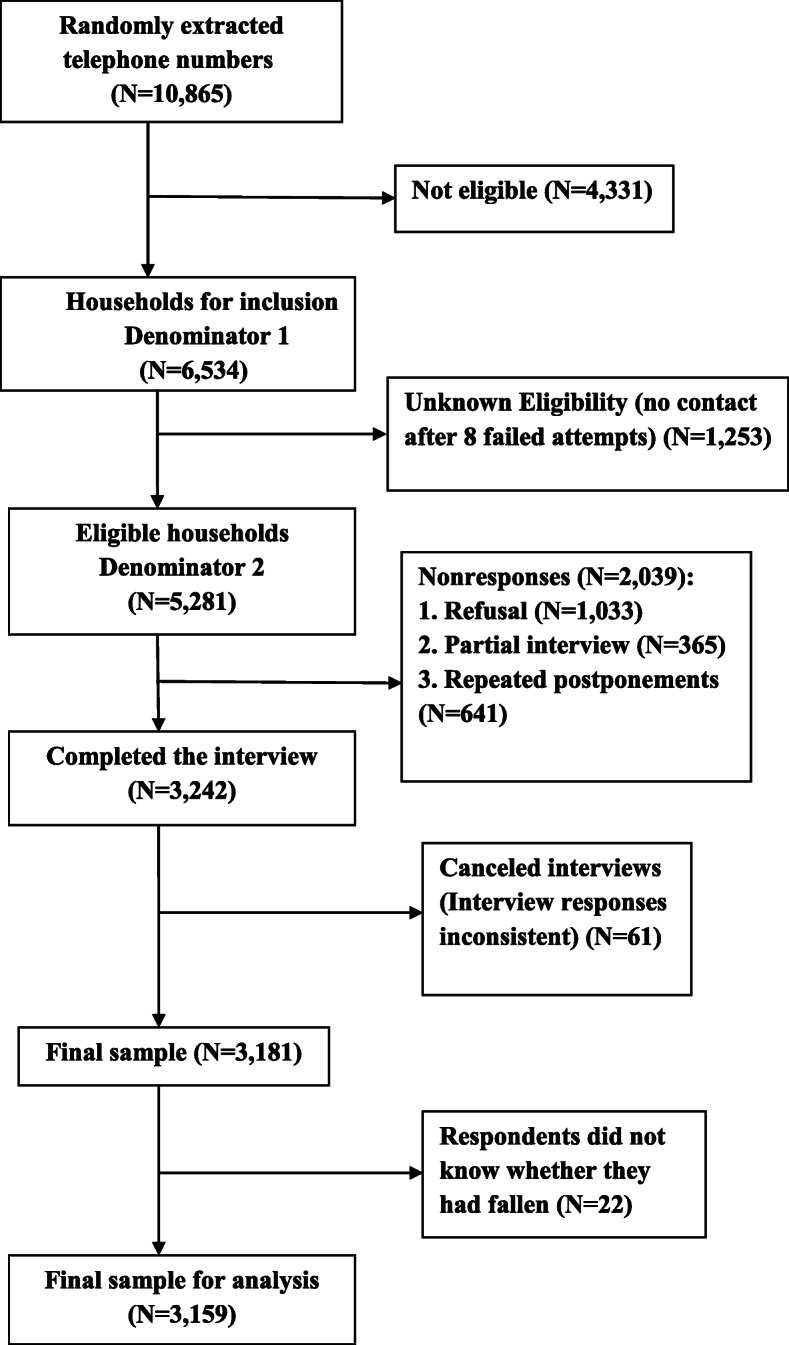
Outcome of household telephone-calls

A random sample of telephone numbers of 10,865 households was extracted: 5000 Jewish households and 5865 Arab households. After excluding non-eligible households and households with unknown eligibility (non-respondents), a total of 5281 households remained; of them 3242 participants completed the survey (Fig. 1). The response rate was 49.6% (if the unknown eligible households were considered, denominator 1) and 61.4% (if the unknown eligible households were not considered, denominator 2). After excluding 61 inconsistent interviews and 22 interviews of respondents who reported not knowing whether they had fallen within the last year, the sample for analysis included 3159 participants (2072 Jews and 1087 Arabs). Basic demographic characteristics are shown in Table 1. The mean age of the participants was 75.11 ± 6.15 years. The majority were women (1809, 57.3%), and married or living with a partner (2160, 69.1%).

**Table 1:** Demographic characteristics of the respondents by whether they fell during the past 12 months

		Falls^a^ in the past 12 months		
Variable	All n, (%)	Yes n, (%)	No n, (%)	*P* value
**Age**				0.001>
65–69	602 (19.1%)	107 (14.2%)	495 (20.6%)	
70–74	907 (28.7%)	173 (23.0%)	734 (30.5%)	
75–79	872 (27.6%)	241 (32.0%)	631 (26.2%)	
≥ 80	778 (24.6%)	232 (30.8%)	546 (22.7%)	
**Gender**				0.001>
Male	1350 (42.7%)	221 (29.3%)	1129 (46.9%)	
Female	1809 (57.3%)	532 (70.7%)	1277 (53.1%)	
**Population group**				0.56
Jewish	2072 (65.6%)	500 (66.5%)	1572 (65.3%)	
Arab	1087 (34.4%)	253 (33.5%)	834 (34.7%)	
**Years of schooling**				0.001>
≤ 12	1753 (56.9%)	458 (62.9%)	1295(55.1%)	
> 12	1327 (43.1%)	270 (37.1%)	1057 (44.9%)	
**Marital Status**				0.001>
Married or living with a partner	2160 (69.1%)	431 (57.7%)	1729 (72.7%)	
Single or widowed	159 (5.1%)	43 (5.8%)	116 (4.9%)	
Separated	805 (25.8%)	273 (36.5%)	532 (22.4%)	

^a^At least one fall in the past 12 months

### Falls

In this survey, 23.8% of the respondents reported falling at least once in the past 12 months. Of them, 55.4% reported falling once, 21.7% twice, 11.1% three times and 11.8% four times or more. Women (70.7%) were more likely to report falling than men (29.3%, *P* < 0.001) (Table 1). The proportion of adults who fell increased with age (*P* < 0.001). There was no significant difference in the reported prevalence of falling between the Israeli Arab and Jewish population groups (*P* = 0.56). Regarding the place of fall, 45.3% had fallen outdoors (streets, parks), 45.5% in their own homes and 9.2% indoors but not in their own house. The majority of the participants (91.1%) reported that they had never received any instruction about fall prevention from their medical care provider. Furthermore, among those who had fallen in the previous year only 11.9% received any fall prevention education. Fall-related fractures were reported by 19.7% of the participants. Arm or elbow fractures accounted for 41.3% of the total fractures, followed by leg or ankle fractures (19.3%). All the known risk factors for falls that were examined were significantly higher among those who fell than among those who did not (Table 2).

**Table 2:** Health characteristics of the study participants by whether they fell in the past 12 months

		Falls^a^ in the past 12 months		
Variable^b^	All n (%)	Yes n (% within fallers)	No n (% within non-fallers)	*P* value
**Difficulties in bathing or dressing**				0.001>
No difficulty	2305 (73.4%)	389 (52.3%)	1916 (80.0%)	
Little difficulty	439 (14.0%)	143 (19.2%)	296 (12.4%)	
Major difficulty/Disabled	396 (12.6%)	212 (28.5%)	184 (7.7%)	
**Difficulties in doing household activities**				0.001>
No difficulty	1811 (58.2%)	260 (35.4%)	1551 (65.3%)	
Little difficulty	556 (17.9%)	144 (19.6%)	412 (17.3%)	
Major difficulty/Disabled	744 (23.9%)	331 (45.0%)	413 (17.4%)	
**Memory and concentration difficulties**				0.001>
No difficulty	1916 (61.9%)	354 (48.8%)	1562 (65.9%)	
Little difficulty	978 (31.6%)	274 (37.8%)	704 (29.7%)	
Major difficulty/Disabled	203 (6.6%)	97 (13.4%)	106 (4.5%)	
**Difficulties in walking**				0.001>
No difficulty	1495 (47.8%)	206 (27.6%)	1289 (54.1%)	
Little difficulty	848 (27.1%)	195 (6.2%)	653 (27.4%)	
Major difficulty/Disabled	721 (23.0%)	306 (41.0%)	415 (17.4%)	
No mobility	66 (2.1%)	39 (5.2%)	27 (1.1%)	
**Impaired vision**, yes, n (%)	862 (27.5%)	276 (37.1%)	586 (24.5%)	0.001>
**Hearing problems**, yes, n (%)	969 (32.5%)	303 (42.5%)	666 (29.4%)	0.001>
**Use of walking aids**, yes, n (%)	592 (19.2%)	263 (36.9%)	329 (13.9%)	0.001>
**Physical activity**				0.001>
Yes, n (%)	1539 (48.9%)	297 (39.7%)	1242 (51.8%)	
No, n (%)	1608 (51.1%)	451 (60.3%)	1157 (48.2%)	
**Morbidity**				
History of heart attack, yes, n (%)	443 (14.1%)	122 (16.4%)	321 (13.4%)	0.046
Cardiac (arrhythmia/chronic heart failure), yes, n (%)	630 (21.1%)	227 (32.9%)	403 (17.6%)	0.001>
History of stroke, yes, n (%)	266 (8.5%)	96 (13.0%)	170 (7.2%)	0.001>
Diabetes mellitus, yes, n (%)	1058 (33.8%)	315 (42.1%)	743 (31.2%)	0.001>
Parkinson, yes, n (%)	30 (1.0%)	16 (2.2%)	14 (0.6%)	0.001
Arthritis, yes, n (%)	572 (18.7%)	203 (28.4%)	369 (15.7%)	0.001>
Depression/Anxiety, yes, n (%)	485 (15.6%)	204 (27.9%)	281 (11.8%)	0.001>
**Use of medications (number)**				0.001>
0	346 (11.0%)	47 (6.3%)	299 (12.4%)	
1–4	1776 (56.3%)	339 (45.1%)	1437 (59.8%)	
5–8	729 (23.1%)	233 (31.0%)	496 (20.6%)	
> 8	306 (9.7%)	133 (17.7%)	173 (7.2%)	
**Type of medications**				
Sleeping medications, yes, n (%)	578 (18.4%)	200 (26.9%)	378 (15.8%)	0.001>
Mood medications, yes, n (%)	351 (11.3%)	148 (20.1%)	203 (8.5%)	0.001>
**Body mass index (BMI** kg/m^2^**)**				0.43
BMI-Underweight (< 18.5)	33 (1.2%)	6 (0.9%)	27 (1.3%)	
BMI-Normal weight (18.5–24.9)	883 (31.4%)	197 (30.2%)	686 (31.8%)	
BMI-Overweight (25.0–29.9)	1234 (43.9%)	282 (43.2%)	952 (44.1%)	
BMI-Obese (≥30)	661 (23.5%)	168 (25.7%)	493 (22.8%)	
**Having fear of falling**, yes, n (%)	1416 (48.2%)	507 (69.8%)	909 (41.4%)	0.001>
**Interruption of activities due to fear of falling**, yes, n (%)	761 (56.0%)	313 (65.2%)	448 (51.0%)	0.001>

^a^At least one fall in the past 12 months

^b^Variables were weighted by population group, age and gender

The multivariate logistic regression analysis (Table 3) found that falls were associated with older age, female gender, major difficulty in bathing or dressing, the use of walking aids, major difficulty in memory and concentration, the use of mood medications, hearing problems, cardiac disease, diabetes mellitus and FOF.

**Table 3:** Adjusted^a^ odds ratios (OR) for associations between risk factors and falls

	Falls in the past 12 months	
Variable	OR	CI 95%
**Age**	1.03	1.01–1.05
**Gender**		
Male	1 (Ref)	–
Female	2.03	1.58–2.61
**Difficulties in bathing or dressing**		
No difficulty	1 (Ref)	–
Little difficulty	1.50	1.07–2.10
Major difficulty/Disabled	2.50	1.64–3.80
**Memory and concentration difficulties**		
No difficulty	1 (Ref)	–
Little difficulty	1.31	1.02–1.69
Major difficulty/Disabled	1.77	1.09–2.88
**Hearing problems** (yes)	1.32	1.03–1.69
**Diabetes mellitus** (yes)	1.35	1.06–1.72
**Cardiac disease** (yes)	1.41	1.08–1.84
**Mood medications** (yes)	1.66	1.18–2.34
**Use of walking aids** (yes)	1.54	1.11–2.15
**Fear of falling** (yes)	1.66	1.29–2.13

^a^Variables that were included in the model but were not statistically significant: population group, education, number of medications, sleep medications, impaired vision, physical activity, arthritis and Parkinson’s disease

### Fear of falling

Fear of falling was reported by 1416 (48.2%) participants and was significantly more prevalent among those who had fallen in the previous year (69.8%) than among those who had not (41.4%, *P* < 0.001). Interruption of activities due to FOF was reported significantly more among fallers (65.2%) than non-fallers (51.0%, *P* < 0.001). Multivariate logistic regression analysis (Table 4) showed that FOF was associated with female gender, major difficulty in bathing or dressing, major difficulty in memory and concentration, the use of sleep medications, cardiac disease, diabetes mellitus, arthritis, the use of walking aids and falls during the last year.

**Table 4:** Adjusted^a^ odds ratios (OR) for the associations between risk factors and fear of falling

	Fear of falling	
Variable	OR	CI 95%
**Gender**		
Male	1 (Ref)	
Female	2.87	2.33-3.53
**Difficulties in bathing or dressing**		
No difficulty	1 (Ref)	
Little difficulty	2.37	1.70-3.31
High difficulty/Disabled	3.14	1.94-5.07
**Memory and concentration difficulties**		
No difficulty	1 (Ref)	
Little difficulty	1.63	1.31-2.02
High difficulty/Disabled	2.81	1.51-5.22
**Sleep medications** (yes)	1.62	1.23-2.13
**Use of walking aids** (yes)	1.83	1.29-2.58
**Diabetes mellitus** (yes)	1.32	1.07-1.63
**Cardiac disease** (yes)	1.65	1.28-2.12
**Arthritis** (yes)	1.57	1.17-2.12
**Falls during the last year** (yes)	1.69	1.31-2.18

^a^Variables that were included in the model but were not statistically significant: population group, age, education, number of medications, mood medications, impaired vision, hearing problems, physical activity and Parkinson’s disease

## Discussion

In this national survey, 23.8% of Israeli community-dwelling people aged ≥65 years reported falling at least once in the past 12 months. The prevalence of falls in a 12-month period reported in the current study is consistent with the prevalence reported among community-dwelling older adults aged ≥65 years in other population-based studies: 20.0% in Canada, 24.4% in the UK and 22.0 and 28.7% in the USA [5, 13–15]. Risk factors that were associated with falls and FOF are consistent with those reported in other studies and include female gender [5, 11], major functional difficulties [16–18], comorbidity [16, 17], the use of walking aids [11, 19, 20] and psychotropic medications [21, 22]. The literature suggests that fall rates may differ between different racial and ethnic groups [23]. In the current study, the prevalence of falls did not differ between the Jewish and Arab participants who account for approximately one-fifth of the total Israeli population.

FOF was reported by nearly half of the participants. FOF usually arises after a fall but can also occur without a history of falls [24]. FOF was significantly more prevalent among participants who had fallen in the previous year (69.8%) but also highly prevalent (41.4%) among those who had not fallen. This finding highlights the importance of screening for FOF in the older population. Indeed, in a systematic review that included 21 studies, the prevalence of FOF ranged from 21 to 85% among community-dwelling older adults, and over 50% of people with FOF did not experience a fall [10].

The current study showed that falls and FOF were each a risk factor for the other. Friedman et al. showed that a person who has one of these factors is at risk for developing the other [24]. The authors showed that a “vicious cycle” exists between falls, FOF, and the many adverse outcomes that can result, such as functional decline, a decrease in quality of life, and institutionalization.

The current study demonstrated a higher rate of interruption of activities due to FOF among those who fell than among those who did not. Delbaere et al. showed that this pattern might fuel fear and avoidance, and cause further deterioration of physical performance and increased risk of falls in the long term [25].

Notably, fall prevention interventions have been shown to reduce both the risk of falling and FOF among community-dwelling older adults [26, 27]. The majority of the participants in this survey (91.1%) reported that they had never received any instruction about fall prevention from their medical care provider. Among those who had fallen in the previous year only 11.9% received any fall prevention education. Healthcare professionals have a major role in increasing awareness about falls and in educating older people about the risk factors associated with falls [26]. The current findings support the importance of the national program for falls prevention whose aims are to identify older people at risk for falls and to develop appropriate falls prevention interventions.

This study has some limitations. First, the data collected were self-reported and subject to recall bias. Some of the participants may have under-reported falls. This would result in a reported prevalence of falls that is lower than the actual rate. Second, our results cannot be generalized to the entire population over 65 years old as the survey did not include people in long-term care facilities who are at higher risk of falls. Third, the cross-sectional study design does not enable determining causal relations of falls or FOF with associated factors. The strength of this study is that it is a national survey that included respondents from the two main population groups in Israel.

## Conclusions

The current findings support the national program for falls prevention and indicate that it should focus not only on falls but also on FOF among the older population. The current findings could assist in the development of specific interventions and prevention strategies to target those at higher risk for falls and FOF.

## Data Availability

Data are available from the authors upon reasonable request and with permission of the Israel Center of Disease Control.

## Abbreviations

FOF: Fear of falling
ICDC: Israeli Center for Disease Control
MOH: Ministry of Health
